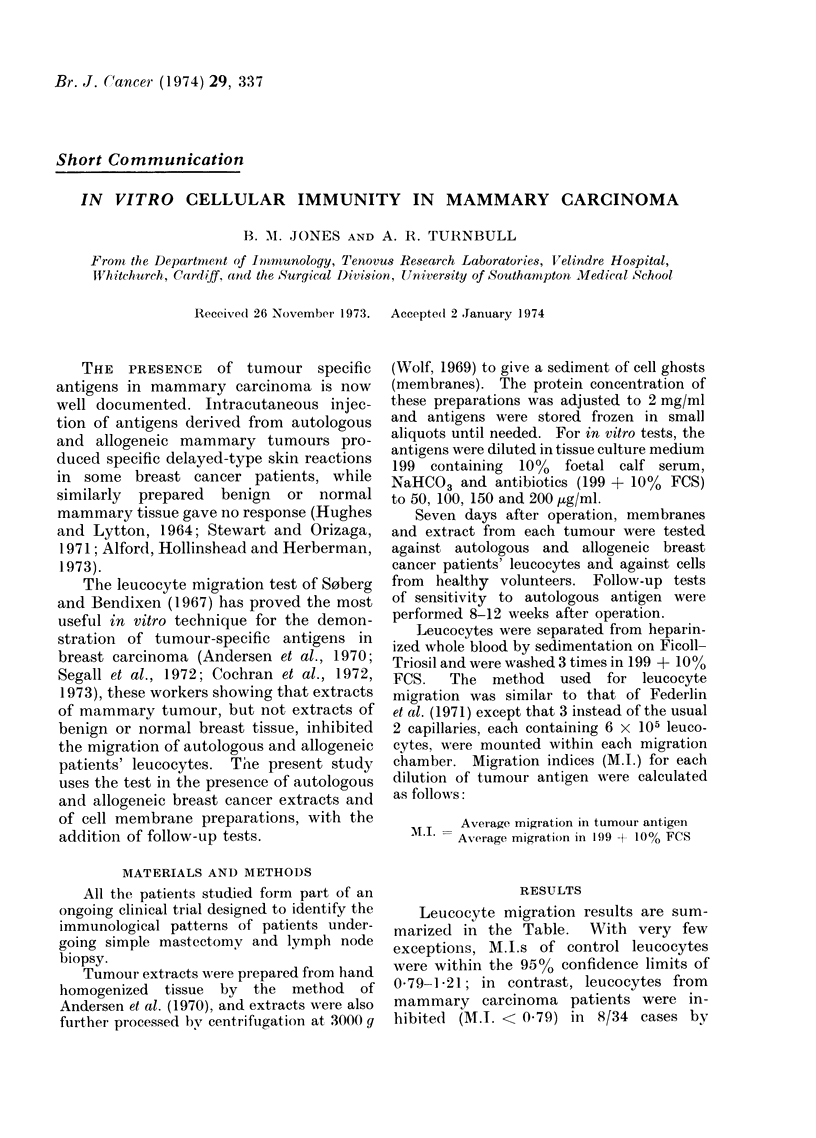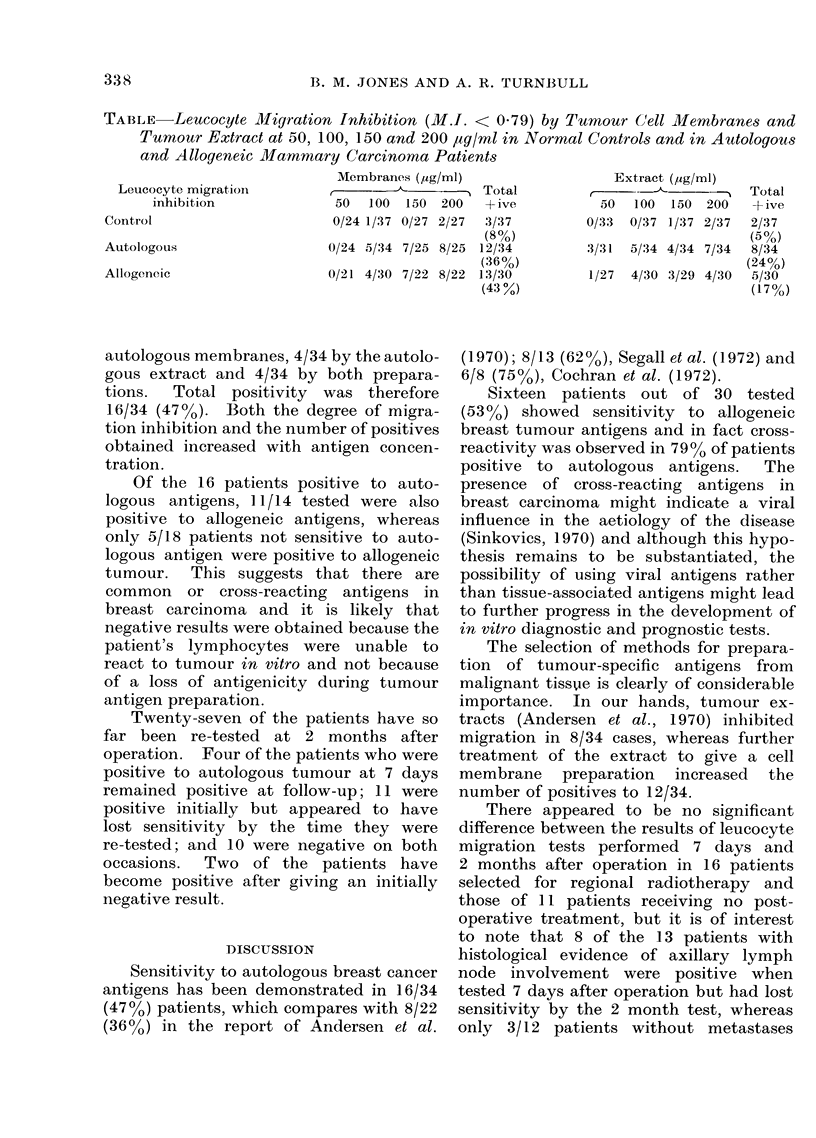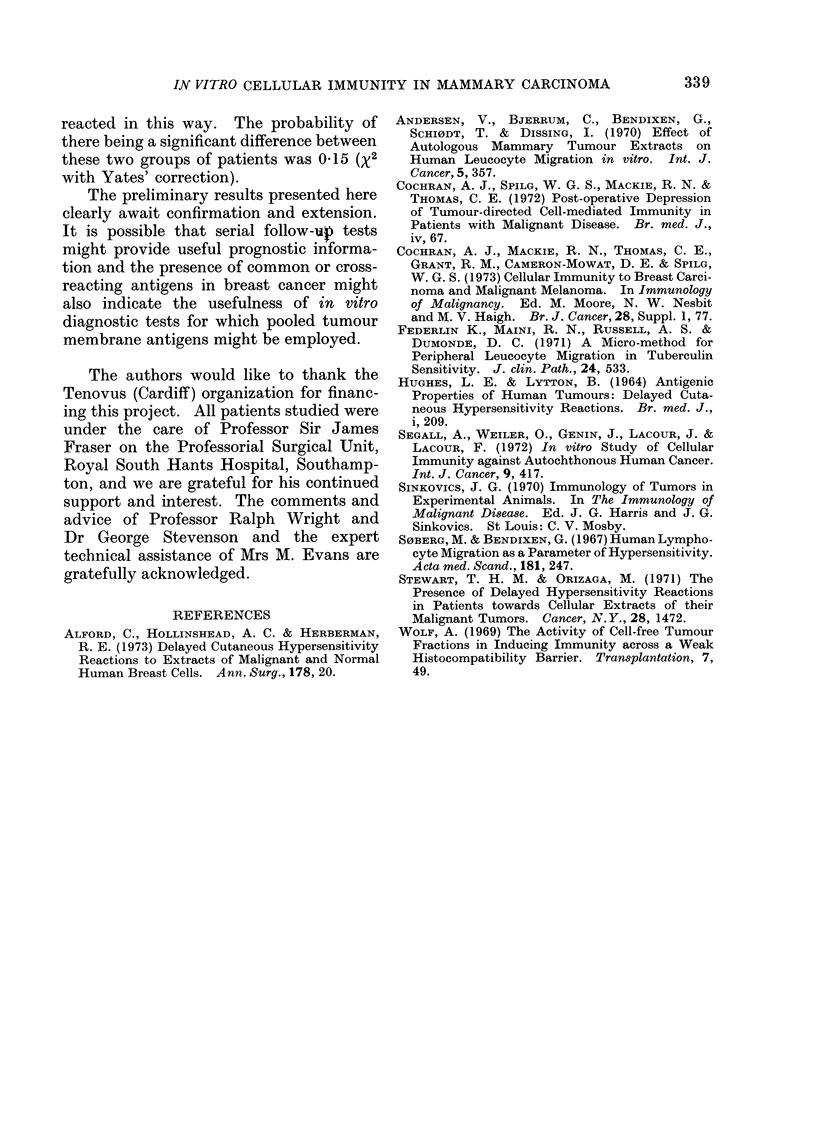# In vitro cellular immunity in mammary carcinoma.

**DOI:** 10.1038/bjc.1974.76

**Published:** 1974-04

**Authors:** B. M. Jones, A. R. Turnbull


Br. J. Cancer (1974) 29, 337

*Short Communication*

### *IN VITRO* CELLULAR IMMUNITY IN MAMMARY CARCINOMA

B. M. JONES AND A. R. TURNBULL

*From the Department of Immunology, Tenovus Research Laboratories, Velindre Hospital, Whitchurch, Cardiff, and the Surgical Division, University of Southampton Medical School*

Received 26 November 1973. Accepted 2 January 1974

THE PRESENCE of tumour specific antigens in mammary carcinoma is now well documented. Intracutaneous injection of antigens derived from autologous and allogeneic mammary tumours produced specific delayed-type skin reactions in some breast cancer patients, while similarly prepared benign or normal mammary tissue gave no response (Hughes and Lytton, 1964; Stewart and Orizaga, 1971; Alford, Hollinshead and Herberman, 1973).

The leucocyte migration test of Søberg and Bendixen (1967) has proved the most useful *in vitro* technique for the demonstration of tumour-specific antigens in breast carcinoma (Andersen *et al.*, 1970; Segall *et al.*, 1972; Cochran *et al.*, 1972, 1973), these workers showing that extracts of mammary tumour, but not extracts of benign or normal breast tissue, inhibited the migration of autologous and allogeneic patients' leucocytes. The present study uses the test in the presence of autologous and allogeneic breast cancer extracts and of cell membrane preparations, with the addition of follow-up tests.

### MATERIALS AND METHODS

All the patients studied form part of an ongoing clinical trial designed to identify the immunological patterns of patients undergoing simple mastectomy and lymph node biopsy.

Tumour extracts were prepared from hand homogenized tissue by the method of Andersen *et al.* (1970), and extracts were also further processed by centrifugation at 3000 *g* (Wolf, 1969) to give a sediment of cell ghosts (membranes). The protein concentration of these preparations was adjusted to 2 mg/ml and antigens were stored frozen in small aliquots until needed. For *in vitro* tests, the antigens were diluted in tissue culture medium 199 containing 10% foetal calf serum, $NaHCO_3$ and antibiotics (199 + 10% FCS) to 50, 100, 150 and 200 μg/ml.

Seven days after operation, membranes and extract from each tumour were tested against autologous and allogeneic breast cancer patients' leucocytes and against cells from healthy volunteers. Follow-up tests of sensitivity to autologous antigen were performed 8–12 weeks after operation.

Leucocytes were separated from heparinized whole blood by sedimentation on Ficoll–Triosil and were washed 3 times in 199 + 10% FCS. The method used for leucocyte migration was similar to that of Federlin *et al.* (1971) except that 3 instead of the usual 2 capillaries, each containing $6 \times 10^5$ leucocytes, were mounted within each migration chamber. Migration indices (M.I.) for each dilution of tumour antigen were calculated as follows:

$$\text{M.I.} = \frac{\text{Average migration in tumour antigen}}{\text{Average migration in 199 + 10\% FCS}}$$

### RESULTS

Leucocyte migration results are summarized in the Table. With very few exceptions, M.I.s of control leucocytes were within the 95% confidence limits of 0·79–1·21; in contrast, leucocytes from mammary carcinoma patients were inhibited (M.I. < 0·79) in 8/34 cases by

TABLE—*Leucocyte Migration Inhibition* (*M.I.* < 0·79) *by Tumour Cell Membranes and Tumour Extract at* 50, 100, 150 *and* 200 *μg/ml in Normal Controls and in Autologous and Allogeneic Mammary Carcinoma Patients*

| Leucocyte migration inhibition | Membranes (μg/ml) | | | | Total +ive | Extract (μg/ml) | | | | Total +ive |
|---|---|---|---|---|---|---|---|---|---|---|
| | 50 | 100 | 150 | 200 | | 50 | 100 | 150 | 200 | |
| Control | 0/24 | 1/37 | 0/27 | 2/27 | 3/37 (8%) | 0/33 | 0/37 | 1/37 | 2/37 | 2/37 (5%) |
| Autologous | 0/24 | 5/34 | 7/25 | 8/25 | 12/34 (36%) | 3/31 | 5/34 | 4/34 | 7/34 | 8/34 (24%) |
| Allogeneic | 0/21 | 4/30 | 7/22 | 8/22 | 13/30 (43%) | 1/27 | 4/30 | 3/29 | 4/30 | 5/30 (17%) |

autologous membranes, 4/34 by the autologous extract and 4/34 by both preparations. Total positivity was therefore 16/34 (47%). Both the degree of migration inhibition and the number of positives obtained increased with antigen concentration.

Of the 16 patients positive to autologous antigens, 11/14 tested were also positive to allogeneic antigens, whereas only 5/18 patients not sensitive to autologous antigen were positive to allogeneic tumour. This suggests that there are common or cross-reacting antigens in breast carcinoma and it is likely that negative results were obtained because the patient's lymphocytes were unable to react to tumour *in vitro* and not because of a loss of antigenicity during tumour antigen preparation.

Twenty-seven of the patients have so far been re-tested at 2 months after operation. Four of the patients who were positive to autologous tumour at 7 days remained positive at follow-up; 11 were positive initially but appeared to have lost sensitivity by the time they were re-tested; and 10 were negative on both occasions. Two of the patients have become positive after giving an initially negative result.

### DISCUSSION

Sensitivity to autologous breast cancer antigens has been demonstrated in 16/34 (47%) patients, which compares with 8/22 (36%) in the report of Andersen *et al.* (1970); 8/13 (62%), Segall *et al.* (1972) and 6/8 (75%), Cochran *et al.* (1972).

Sixteen patients out of 30 tested (53%) showed sensitivity to allogeneic breast tumour antigens and in fact cross-reactivity was observed in 79% of patients positive to autologous antigens. The presence of cross-reacting antigens in breast carcinoma might indicate a viral influence in the aetiology of the disease (Sinkovics, 1970) and although this hypothesis remains to be substantiated, the possibility of using viral antigens rather than tissue-associated antigens might lead to further progress in the development of *in vitro* diagnostic and prognostic tests.

The selection of methods for preparation of tumour-specific antigens from malignant tissue is clearly of considerable importance. In our hands, tumour extracts (Andersen *et al.*, 1970) inhibited migration in 8/34 cases, whereas further treatment of the extract to give a cell membrane preparation increased the number of positives to 12/34.

There appeared to be no significant difference between the results of leucocyte migration tests performed 7 days and 2 months after operation in 16 patients selected for regional radiotherapy and those of 11 patients receiving no post-operative treatment, but it is of interest to note that 8 of the 13 patients with histological evidence of axillary lymph node involvement were positive when tested 7 days after operation but had lost sensitivity by the 2 month test, whereas only 3/12 patients without metastases reacted in this way. The probability of there being a significant difference between these two groups of patients was 0·15 ($\chi^2$ with Yates' correction).

The preliminary results presented here clearly await confirmation and extension. It is possible that serial follow-up tests might provide useful prognostic information and the presence of common or cross-reacting antigens in breast cancer might also indicate the usefulness of *in vitro* diagnostic tests for which pooled tumour membrane antigens might be employed.

The authors would like to thank the Tenovus (Cardiff) organization for financing this project. All patients studied were under the care of Professor Sir James Fraser on the Professorial Surgical Unit, Royal South Hants Hospital, Southampton, and we are grateful for his continued support and interest. The comments and advice of Professor Ralph Wright and Dr George Stevenson and the expert technical assistance of Mrs M. Evans are gratefully acknowledged.

### REFERENCES

ALFORD, C., HOLLINSHEAD, A. C. & HERBERMAN, R. E. (1973) Delayed Cutaneous Hypersensitivity Reactions to Extracts of Malignant and Normal Human Breast Cells. *Ann. Surg.*, **178**, 20.

ANDERSEN, V., BJERRUM, C., BENDIXEN, G., SCHIØDT, T. & DISSING, I. (1970) Effect of Autologous Mammary Tumour Extracts on Human Leucocyte Migration *in vitro*. *Int. J. Cancer*, **5**, 357.

COCHRAN, A. J., SPILG, W. G. S., MACKIE, R. N. & THOMAS, C. E. (1972) Post-operative Depression of Tumour-directed Cell-mediated Immunity in Patients with Malignant Disease. *Br. med. J.*, iv, 67.

COCHRAN, A. J., MACKIE, R. N., THOMAS, C. E., GRANT, R. M., CAMERON-MOWAT, D. E. & SPILG, W. G. S. (1973) Cellular Immunity to Breast Carcinoma and Malignant Melanoma. In *Immunology of Malignancy*. Ed. M. Moore, N. W. Nesbit and M. V. Haigh. *Br. J. Cancer*, **28**, Suppl. 1, 77.

FEDERLIN K., MAINI, R. N., RUSSELL, A. S. & DUMONDE, D. C. (1971) A Micro-method for Peripheral Leucocyte Migration in Tuberculin Sensitivity. *J. clin. Path.*, **24**, 533.

HUGHES, L. E. & LYTTON, B. (1964) Antigenic Properties of Human Tumours: Delayed Cutaneous Hypersensitivity Reactions. *Br. med. J.*, i, 209.

SEGALL, A., WEILER, O., GENIN, J., LACOUR, J. & LACOUR, F. (1972) *In vitro* Study of Cellular Immunity against Autochthonous Human Cancer. *Int. J. Cancer*, **9**, 417.

SINKOVICS, J. G. (1970) Immunology of Tumors in Experimental Animals. In *The Immunology of Malignant Disease*. Ed. J. G. Harris and J. G. Sinkovics. St Louis: C. V. Mosby.

SØBERG, M. & BENDIXEN, G. (1967) Human Lymphocyte Migration as a Parameter of Hypersensitivity. *Acta med. Scand.*, **181**, 247.

STEWART, T. H. M. & ORIZAGA, M. (1971) The Presence of Delayed Hypersensitivity Reactions in Patients towards Cellular Extracts of their Malignant Tumors. *Cancer, N.Y.*, **28**, 1472.

WOLF, A. (1969) The Activity of Cell-free Tumour Fractions in Inducing Immunity across a Weak Histocompatibility Barrier. *Transplantation*, **7**, 49.